# Supplementary material for: Local stimulation of osteocytes using a magnetically actuated oscillating beam
Source: PLoS One. 2020 Jun 29;15(6):e0235366. doi: 10.1371/journal.pone.0235366 (PMC7323988; doi:10.1371/journal.pone.0235366)
Supplement: Attachment — Submitted filename: Response to Reviewer Comments Final v3.docx [file pone.0235366.s003.docx]

Review Summary

Based on the comments provided by the reviewers, we have modified our manuscript to provide clarification on the issues raised. These changes focused primarily on justifying the shear stress levels that were generated and how we quantified these values. We also provided an explanation regarding the duration of the experiment, as well as the confluence level used during these experiments. We have also provided clarification for the number of experimental trials that we used to collect our data. We also added a paragraph to the introduction in order to highlight other techniques used for cell manipulation and their advantages and limitations.

**Reviewer #1:** This paper describes the fabrication of a magnetically actuated oscillating beam device for the local cellular stimulation. They validate their device by measuring Ca production from osteocytes upon stimulation. The paper is interesting, and they have performed a thorough characterization of their device; however, there are too many issues that need to be addressed before the manuscript is ready for publication.

My recommendation is that the manuscript deserves a major revision in its current form, and it should be rewritten and re-submitted.

If the authors are prepared to undertake the work required, I would be pleased to reconsider my decision, although this does not imply that the paper will be accepted with certainty in the event that the authors decide to revise and resubmit the work.

1. This manuscript needs a revision for grammar and clarity: e.g., fluorescent microscope (fluorescence microscope). The magnetic physics was (The magnetic physics were) The Couette model consists of flow….., The fluid velocity at different vertical distances from the beam (as shown by Eq. 7) are plotted (is),…. etc.

Thank you very much for your comment! We have undergone a more thorough editing of our manuscript and fixed all the grammar and clarity issues.

2. Different formatting in the references is used. Please use a uniform format. See (4,5) and (9),(10) for instance.

Thank you very much for the comment, we have re-formatted all the references according to PLOS One guidelines.

3. In the introduction section a brief revision about optical, electrical and magnetic non-contact cell manipulation should be included to give the readers a better understanding of the field.

We agree with the reviewer, and the following paragraph was added to the introduction.

Page 3, Lines 62-72

‘While local stimulation of cells has been attempted in the past [1], no study has attempted to quantify the shear stress gradient that can be generated through local non-contact cell manipulation. Contact cell manipulation involves direct physical contact between the cell wall and external tools or forces whereas non-contact cell manipulation is the indirect manipulation of cells through remote actuation methods such as magnetic fields [20], acoustics [4–6], fluid forces [7,8], and dielectrophoresis [9–11]. Contact manipulation allows for precise control of single cells using techniques such as optics or optical tweezers [12–14], magnetic fields [15–17] or bio-actuation [18,19]. These techniques can be embedded into a microfluidic chip and allow for cutting, injecting and stimulation of individual cells with a high degree of selectivity and accuracy [20–22]. Non-contact cell manipulation has the capability to manipulate a larger number of cells in a multitude of environments but loses some of the accuracy and specificity of contact manipulation. They are, however, less likely to damage cells as a result. Magnetically actuated microrobotic tools have been used in the past for cell manipulation tasks such as transportation [2,3] or proposed for other biomedical applications [23].”

4. The introduction is too short to give a good feedback about the current state-of-the art. The talk about AFM but, in addition, a description about the use of magnetic and optical tweezers in order to exert controlled mechanical stimulation on cells should be included.

Thank you for your comment! The following paragraph was added to the introduction.

Page 3, Lines 62-72

‘While local stimulation of cells has been attempted in the past [1], no study has attempted to quantify the shear stress gradient that can be generated through local non-contact cell manipulation. Contact cell manipulation involves direct physical contact between the cell wall and external tools or forces whereas non-contact cell manipulation is the indirect manipulation of cells through remote actuation methods such as magnetic fields [20], acoustics [4–6], fluid forces [7,8], and dielectrophoresis [9–11]. Contact manipulation allows for precise control of single cells using techniques such as optics or optical tweezers [12–14], magnetic fields [15–17] or bio-actuation [18,19]. These techniques can be embedded into a microfluidic chip and allow for cutting, injecting and stimulation of individual cells with a high degree of selectivity and accuracy [20–22]. Non-contact cell manipulation has the capability to manipulate a larger number of cells in a multitude of environments but loses some of the accuracy and specificity of contact manipulation. They are, however, less likely to damage cells as a result. Magnetically actuated microrobotic tools have been used in the past for cell manipulation tasks such as transportation [2,3] or proposed for other biomedical applications [23].”

5. Figure 1f. How many replicas were performed?

The cell death measurement was conducted on 3 separate magnetic samples. We have included this information in the figure caption of Fig.1f.

6. There is an error in Figure 2 caption. There is not Figure 2f. They are repeating the same Figure 1f caption.

Sorry for the mistake. We have removed the erroneous figure caption for Fig.2f.

7. The authors say: “However, the magnitude of the shear stress must be greater than 0.5Pa to result in cell stimulation(6)”. But in ref 6 at 1Hz, differences in cell stimulation compared untreated controls takes place after 2h of stimulation. So timing is important, and they authors of this current manuscript should include an explanation about the exposition times used in their work (10 min for the intracellular Ca imaging) and the rapid cellular response observed compared to other works. Why?

This is a good point. In our experimental setup, we have chosen to use intracellular calcium concentration increase as a marker for early cellular response to mechanical loading. It has been known that intracellular calcium increase is one of the major early events in cellular response to biochemical challenges. Intracellular calcium is an important second messenger which can trigger various downstream pathways and lead to changes in cytoskeletal organization, mRNA expression, and protein expression. We followed previous protocols published in literature that used up to 10 min of mechanical loading and measuring intracellular calcium concentration as short-term outcome measurements of osteocyte response to shear stress[24–26].This is only an indicator for the beginning of osteocyte’s response to mechanical loading. In ref (6), the authors used mRNA as a marker of osteocyte response to mechanical loading. Due to the biological mechanism of changing mRNA levels, it typically takes much longer than immediate signals such as calcium influx, hence in ref (6) the authors used longer stimulation times for their studies. While other markers such as mRNA and soluble proteins are also important factors to consider, they typically require longer analysis or culturing periods. We plan on conducting this analysis in the next generation devices where the microfluidic platform will be designed to be more adaptable to these longer-term analytical techniques. We have modified the following segment to our methods section to clarify this:

Page 9, Lines 205-209

“Calcium imaging protocols are based on previously existing studies in literature [51–53]. Briefly, MLO-Y4 cells are stained with Fura-2 AM intracellular calcium dye (ThermoFisher Scientific, USA) for 45 min at room temperature in darkness. After rinsing with phosphate-buffered saline (PBS, Sigma-Aldrich, USA) and resting on a heated imaging stage for 15 min, experimental slides seeded with stained cells are imaged by an Nikon Eclipse fluorescence microscope for 1-2 minutes before the magnetic field is turned on to oscillate the beam for up to 10 minutes.”

We have also modified the section below to specify the reason behind the shear stress level used:

Page 5, Line 126-132

“The device is placed in the center of the workspace such that the magnetization direction of the beam is perpendicular to the external magnetic flux density. An oscillating magnetic flux density of 10 mT at 1 Hz (Fig 4b) causes the beam to oscillate in the x-y plane [45] that results in fluid shear stress on the cell surface. From previous literature, commonly accepted range of fluid shear stress sensed by osteocytes in the lacuna-canaliculi network is estimated to be around 0.8 – 3 Pa [9], with our previous work showing a response from MLO-Y4 osteocytes at a minimum of 0.5 Pa [6]. Hence, the magnitude of the shear stress must be greater than 0.5 Pa to result in cell stimulation [6], which occurs for very small beam-surface gap heights.”

8. Why the fluid viscosity used in equation 2 is constant? Can it vary depending on the Ca production upon stimulation? The authors say: “a release of nutrients and chemicals from the stimulated cells”. So, does the medium change in composition depending on the stimulation? Have they calculated the viscosity of the media containing 4.6 mg/mL Dextran (500k MW) in their calculations??? An explanation is needed.

Thank you. We have edited our manuscript to reflect that the emphasis is on the release of signalling molecules instead of said nutrients. These signalling factors play an important role in intercellular communication. Specifically, we are using intracellular calcium concentrations to measure for cell response to loading. The concentrations of these signalling factors are typically very low, and have a minimal effect on the viscosity of the medium compared to other components such as sugar and fetal bovine serum. For example, signalling protein expression from osteocytes *in vitro* are in the range of 20 ng/mL [27], while concentrations from cell media components such as glucose is 1 g/mL [28], effectively 10^9^ higher concentration than cell secreted signals.

The reason Dextran was added to the medium was to increase the viscosity of the fluid so that a stronger shear stress can be applied to the cells via the magnetic actuator. From previous work [29], it has been shown that the addition of dextran to the media does not affect osteocyte mechano-sensitivity to fluid shear stress. From this previous work, it has been shown the viscosity of the media is constant at around 4.73 x 10^-3^ Pa s despite changing shear rates.

Page 10, Line 226-227

“a release of signalling factors from the stimulated cells in the LSR cross talk with cells outside the LSR that lead to their response through intercellular communication.”

9. The authors say: “a release of nutrients and chemicals from the stimulated cells” Why a cell is going to release nutrients to the neighboring cells? Do neighboring non-stimulated cells scavenge for other nutrients to support their metabolism? An explanation is needed.

Thank you. This was a poorly-phrased sentence on our part. We have corrected the statement in our manuscript to read “a release of signalling factors from the stimulated cells in the LSR cross talk with cells outside the LSR that lead to their response through intercellular communication” (Page 10, Line 226-227). Our goal here is to explain that a release of signalling factors can affect neighbouring osteocytes under mechanical loading conditions.

10. The authors say: Despite the relatively far distance between the LSR and responding cells further away (100-250 μm) from the magnetic actuator, extracellular vesicles could play a key role in delivering signals at distances beyond the immediate surroundings of the LSR(18,29). They could collect exosomes from nonstimulated and stimulated cells and running a proteomic analysis to validate their hypothesis. Also, to know by a simple western blotting if the number of exosomes is higher upon stimulation. If not, I do not understand why that sentence in this manuscript.

For our proof-of-concept studies, we are currently working with an experimental prototype for our validation experiments. The LSR is a very small area on a standard glass slide, and we do not have the means to accurately extract the exosomes from stimulated cells versus other non-stimulated cells on the same slide. It is only our speculation that exosomes can play a role here; since signal propagation between cells can go through different pathways, we are currently unable to pinpoint the exact method for signal propagation outside of the LSR. Our future work includes development of an enclosed microfluidic device where smaller chambers housing multiple magnetic actuators will be used to allow for easier isolation of condition media from stimulated cells. We are hoping to conduct a more thorough analysis of osteocyte response to the magnetic actuator once we have the new devices fabricated. We have made the following clarification in our manuscript:

Page 10, Lines 249-253

“It is interesting to note that despite the relatively far distance between the LSR and responding cells further away (100-250 µm) from the magnetic actuator, previous work has reported that extracellular vesicles could play a key role in delivering signals at a distance [18,51]. Since *in vitro* studies using cell indentation tools have shown it is difficult for calcium signals alone to propagate intracellular signalling beyond its neighbouring cells [17], we speculate that exosomes could be a potential explanation for cells responding outside of LSR.”

11. In Figure 5b scattered cells are shown. Do you need have confluent cells as in a physiological media to draw conclusions? Does the cell number play a role in Ca response? Experiments with different cell numbers are needed.

This is a very good point. For all of our imaging experiments, we have been using approximately 80% cell confluence on our glass slides. This follows common culturing practices of osteocytes found in literature [28] since lower cell confluence could lead to potential osteocyte mutations and change cell behaviours. We chose the standard cell confluence level to reduce the number of factors that would affect osteocyte response to shear stress as we are testing out our preliminary platform. This confluence level will help us understand the basic response rate and response patterns when stimulated with the magnetic actuator. Furthermore, at sub-full confluence, osteocyte dendrites would have more room to extend and sense oncoming shear stress. Future experiments with varying levels of cell confluence will allow us to measure parameters such as minimum distance required for signal propagation, and whether osteocytes can still propagate calcium signals at full confluence with minimal dendritic extensions. We plan on conducting these experiments in the future in our new encased microfluidic devices once they are fabricated.

**Reviewer #2:** The authors have engineered a microfluidic device where mechanical stimulations can be applied on cells seeded in a monolayer on the bottom of the devices. For this a magnetically actuated oscillating beam was developed to generate shear stresses on the cells thanks to the oscillatory movements of the beam. Microfluidic devices are complex engineered system that can be compromised at any step of their fabrication which make this work notable. However some concerns regarding the validation can be raised.

1- One major comment on this study, is the few statistical analysis provided regarding the capacity of the devices to stimulate osteocyte-like cells (MLO-Y4) and to induce intracellular calcium signalling. Indeed, many details are lacking specifically in the materials and methods section, ie the number of independent experiments that have been performed and that worked, and if any statistical tests were performed to compare the results of calcium signalling. In the current state of the article, the results are presenting data from only 1 trial since 39 cells were recorded in a video from a single run and are shown in the figure 5 which does not represent the 3 trials that are informed in the caption. In addition for the figure 5c the coefficient of regression is only available in the caption and is not discussed.

Thank you very much for your comment! For our experiments in Fig 5, we have conducted 3 trials that yielded 39 total responding cells. For figure 5, we have combined the results from multiple trials and plotted all responding cells on a single graph (Fig 5a). This was done by taking the relative position of the cell with respect to a corner of the magnetic actuator. This relative position allowed us to also evaluate how cell response varies with distance from the magnetic actuator. The image shown in Fig 5b is a representative image of what the recording window looks like as osteocytes are stained with live calcium reporter. We have modified the figure caption for Fig 5 to make this clearer.

Page 10, Lines 237-244

**“Fig 5: Osteocyte calcium response under magnetic actuator stimulation.** a) The shear stress map obtained using Eq. 6 with velocities obtained from finite element simulations is overlaid with the locations of all responding cells from multiple trials that are coded with the time it takes to respond. The dotted line shows the typical viewing window in experiments and the range of the beam oscillation is also overlaid on the map. b) A representative image obtained of the Fura-2 AM stained cells along with the beam is shown which corresponds to the dotted lines on the shear stress map. Scale bar = 50 µm. c) A plot of the cell response time vs distance from the tip of the beam with a linear regression performed is shown, showing an increase in response time for cells further away from the stimulated region. A total of 3 trials and 39 cells were recorded, with an r^2^ value of 0.59 for the linear regression.”

We have added a comment on the linear coefficient obtained between response time and distance:

Page 10, Lines 230-233

“The linear coefficient between these two variables is calculated to be 0.59, demonstrating a trend between distance and response time. We believe a higher coefficient can be achieved once we adopt the experimental setup in an enclosed microfluidic device, where we will have more control over the gap height between the osteocytes and the magnetic beam, as well as device fabrication and thus the reproducibility of the setup.”

2- Osteocytes are the orchestrator of bone homeostasis and are mechanosensitive cells. What is the minimum of flow subjected on the osteocytes that can induce a response? What is the range of shear stress in the lacunocanalicular network in healthy condition?

The following passage was added to the manuscript to clarify our choice of shear stress level.

Page 6, Lines 128-132

“From previous literature, commonly accepted range of fluid shear stress sensed by osteocytes in the lacuna-canaliculi network is estimated to be around 0.8 – 3 Pa [9], with our previous work showing a response from MLO-Y4 osteocytes at a minimum of 0.5 Pa [6]. Hence, the magnitude of the shear stress must be greater than 0.5 Pa to result in cell stimulation [6], which occurs for very small beam-surface gap heights.”

3- In the FEA, there is no indication of any turbulence induced in the fluid outside of the LSR due to the oscillatory movement of the beam. What str the authors thought about the flow induced outside this area? Could this induce the oscillation of calcium observed in cells far from the beam?

The flow and hence shear stress induced by the oscillating beam decays very quickly outside the LSR. While we have some streamlines outside this region, we do not actually have turbulence according to the calculated Reynold’s number. The authors still postulate that stimulation outside this region is a result of either repeated exposure over a long period of time or due to a nutrients exchange. The following text was added to the manuscript in order to address this comment:

Page 9, Lines 218-222

“We also observed cells being stimulated outside of the LSR where the shear stress magnitude was below the threshold required for cell stimulation. We have two hypotheses as to why this is the case; first, this could be the result of some form of cell response due to prolonged low magnitude shear stress, or secondly, a release of signalling factors from the stimulated cells in the LSR cross talk with cells outside the LSR that lead to their response through intercellular communication.”

4- It is not clear what is the timescale to have a stable flow. And how this can be compared to other devices inducing fluid flow to study calcium signalling in vitro?

At t ~ $\frac{h^{2}}{\nu}$, steady Couette flow is expected to have been reached (fully developed). This is dependent on only the gap height ($h$) and the kinematic viscosity ($\nu$) of the media.

$$t=\frac{h^{2}}{\nu}=\frac{h^{2}\rho}{\mu}=\frac{\left( 5\text{x}{10}^{-6} \right)^{2}\left( 1000 \right)}{4.7x{10}^{-3}}=5.3\text{x}{10}^{-6} \text{s}$$

This is further confirmed below by two simulations that the authors ran for 1 second (1 cycle) and 10 seconds (10 cycles) shown in the figures below and the differences between the two were negligible. Other devices have fully developed flow on the same time scale, but the main difference is that they are able to induce larger shear stresses due to larger plates since they are doing it globally instead of in localized regions as this device proposes to do. The following text was added to the manuscript to convey this to the readers:

Page 9, Lines 211-214

“In Fig 5a, we have plotted the maximum shear stress map resulting from the oscillation of the beam over 10 cycles (the flow has stabilized within 1 cycle as differences between the 1 cycle and 10 cycle simulations appear negligible) in the 1 mm square region of interest around the beam tip”

5- Additional introduction on microfluidics and the fluid flow induced would allow to understand what are the advantages of this device compared to others.

Thank you very much for your comment! The following paragraph was added to the introduction in order to illustrate other devices that have been used and their limitations.

Page 3, Lines 62-72

‘While local stimulation of cells has been attempted in the past [1], no study has attempted to quantify the shear stress gradient that can be generated through local non-contact cell manipulation. Contact cell manipulation involves direct physical contact between the cell wall and external tools or forces whereas non-contact cell manipulation is the indirect manipulation of cells through remote actuation methods such as magnetic fields [20], acoustics [4–6], fluid forces [7,8], and dielectrophoresis [9–11]. Contact manipulation allows for precise control of single cells using techniques such as optics or optical tweezers [12–14], magnetic fields [15–17] or bio-actuation [18,19]. These techniques can be embedded into a microfluidic chip and allow for cutting, injecting and stimulation of individual cells with a high degree of selectivity and accuracy [20–22]. Non-contact cell manipulation has the capability to manipulate a larger number of cells in a multitude of environments but loses some of the accuracy and specificity of contact manipulation. They are, however, less likely to damage cells as a result. Magnetically actuated microrobotic tools have been used in the past for cell manipulation tasks such as transportation [2,3] or proposed for other biomedical applications [23].”

Minor comments

1- In the Introduction, there is a confusion in citing McGarrigle et al 2016. In this article, the authors did not quantify intracellular calcium signalling and no fluid flow systems were used. Is there a confusion with the article from Deepak et al 2017? Please make sure all the other references are cited appropriately.

Thank you for your comment! The citation was indeed for the article you have pointed out regarding Ca+ oscillation under estrogen deficiency conditions. We have corrected the error in our citation and looked through other citations to make sure similar errors are not in place.

2- Some details are lacking in the materials and methods ie the passage of the cells when seeded on the devices.

We have made the following edits to our cell culture protocols to include more details.

Page 9, Lines 196-203

MLO-Y4 osteocytes (courtesy of Dr. Bonewald, Indiana University School of Medicine) are cultured in growth media composed of 2.5% calf serum (CS, Gibco, USA), 2.5% fetal bovine serum (FBS, Gibco, USA), 1% penicillin-streptomycin (PS, Gibco, USA), and 94% Alpha Minimum Essential Medium (MEM) (WISENT, Canada). Cells are seeded during passage 29 at 105 cells per 100 mm diameter collagen-coated (0.15 mg/ml Type I collagen (Corning, USA)) culture dishes and expanded until they achieve 80% confluency. The cells are then transferred onto collagen-coated experimental slides (75x25 mm) at a density of 500k cells per slide for overnight incubation till they reach 80% confluence again before imaging. MLO-Y4 cells are passaged between P29 and P35 while maintained in an incubator at 37 ˚C and 5% CO2. Cell death was quantified using Trypan Blue Stain (Sigma-Aldrich, USA) and counted under a standard light microscope.

3- What is the maximum duration that the system was ran to induce an oscillatory shear stress?

The magnetic field was turned on during experiments for between 2 – 10 minutes in order to induce oscillatory shear stress. Response times for cells were based on a multitude of factors such as gap height and deflection. The total experiment was longer since baseline data was collected before and after the experiment. We have added the following text to our manuscript:

Page 9, Lines 199-202

“After rinsing with phosphate-buffered saline (PBS, Sigma-Aldrich, USA) and resting on a heated imaging stage for 15 min, experimental slides seeded with stained cells are imaged by a Nikon Eclipse fluorescence microscope for 1-2 minutes before the magnetic field is turned on to oscillate the beam for up to 10 minutes.”

4- It would improve the manuscript to add in the discussion a sentence about the absence of control conditions where the cells are subjected to the magnetic field and not to shear stress.

We have done a trial control previously measuring calcium response under the magnetic field without the actuator, and we did not observe any calcium response from the cells. We have made the following edit to our manuscript to reflect our observation.

Page 9. Lines 221-224

“A sample microscope view of the beam and osteocytes is shown in Fig 5b. The maximum shear stress occurs in small areas around the beam oscillation which we refer to as the ‘local stimulation region’ (LSR). We see that cells in and around the LSR are stimulated. This is confirmed to be responses to the magnetic actuator, as cells seeded in the magnetic field without the actuator elicited no quantifiable calcium response.”

**Reviewer #3:** Figures are schematic, illustrative and well captioned. However, they are quite pixelated (especially figures 1, 2 and 3). Graphics could be acceptable in smaller size, but it would be better to see them in higher quality.

Thank you for catching this. This was an export error and the issue has been fixed.

Line 112: “shear stress must be greater than 0.5 Pa to result in cell stimulation”. This is good data to set the experiment but a reference is missed regarding physiological value (or range of values) of shear stress that osteocytes may be exposed to in lacuno-canicular system. Indeed, in lines 19-21 (abstract) you claim that the beam generates the fluid shear stress encountered in vivo but you did not indicate that value. Related to the former comment, in line 61: you set a frequency of 1Hz in order to apply fluid shear stress to the cells. Have you tested different frequencies to get a wider scope of shear stress?

We did not test different frequencies for set of experiments but plan to add this in when we move onto our microfluidic platform. Increasing the frequency is a quick and easy way to increase the fluid velocity and thus the shear stress. This should theoretically increase the LSR region and stimulate more cells as well result in a quicker response time. However, in order to maintain physiologically relevant frequencies such as those experienced during daily tasks or exercise, we would be limited to frequencies under 5 Hz.

We have added the following modification to the manuscript for clarification:

Page 6,

“From previous literature models and predictions, commonly accepted range of fluid shear stress sensed by osteocytes in the lacuna-canaliculi network is estimated to be around 0.8 – 3 Pa [30], with our previous work showing a response from MLO-Y4 osteocytes at a minimum of 0.5 Pa[31].”

Line 174: Cell density is reported to be a key parameter in 'in vitro' models. The density seeded over the experimental slides is (500k cells / (7.5cm*2.5cm); (line179)) ≈ 27.000 cells/cm2. It is a value that is not compared with physiological osteocyte density in murine lacuno-canicular system. Did you check results of calcium responses with different cell densities?

For all of our imaging experiments, we have been using approximately 80% cell confluence on our glass slides. This is different from our seeding density, as we grow our cells to 80% confluence before imaging. This follows common culturing practices in literature [28]. We modified our manuscript below to clarify this:

Page 9, Lines 198-201

“Cells are seeded during passage 29 at 105 cells per 100 mm diameter collagen-coated (0.15 mg/ml Type I collagen (Corning, USA)) culture dishes and expanded until they achieve 80% confluency. The cells are then transferred onto collagen-coated experimental slides (75x25 mm) at a density of 500k cells per slide for overnight incubation till they reach 80% confluence again before imaging.”

We have not yet considered varying cell density and seeing if that has an effect on calcium response. Models from *in vivo* samples have shown an approximate osteocyte density of 72k cells/mm^3^ [32], whereas 80% confluence is equivalent to approximately 3200 cells/mm^2^ (approximating 20 µm cell diameters [33]). Assuming a 20 µm slice, *in vivo* cell density would be around 1440 cells/mm^2^, which is in the same order of magnitude as 80% confluence on glass slides. However, in the future we do plan on looking at cell density in our new encased microfluidic devices once they are fabricated.

Line 186: you have stimulated the culture for 10 min. Afterwards you indicate that “With a prolonged stimulation time, it is possible that the concentration of signaling molecules increased to a threshold level capable of generating a comparable cellular response as fluid shear stress”. Have you tested your experiment for longer times to check this possibility?

For our preliminary validation experiments, due to our current prototype setup for the magnetic actuator device, it is difficult to keep osteocytes in a healthy state and with sufficient nutrients without disrupting the imaging process. Therefore, we have not attempted any imaging experiment longer than 10 min. For our future work, we plan on designing and fabricating an enclosed microfluidic system with media perfusion that will allow us to conduct experiments for longer durations and time periods.

Line 187: “growth media supplemented with Dextran to achieve the needed shear stress without significantly increasing the size of the beam”. It is not completely clear the reason why Dextran is needed to achieve that purpose. It may be also helpful to show a fluorescent picture of the culture.

As with increasing frequency, increasing the viscosity of the media is an easy way to increase the shear stress experienced by cells. This is because shear stress is directly proportional to the viscosity of the media. This has been clarified in the text. Previous work has shown adding dextran will not affect osteocyte response as long as Reynolds number is kept constant [29].

Page 9, Lines 204-205

“This results in an increase in the viscosity of the media which is directly proportional to shear stress.”

You define LSR (local stimulation region) as areas in and around the beam oscillation (line 199). Figure 5, both images “a” and “b”, are useful pictures that ease the understanding of the results you provide. It would be helpful, though, to indicate on both figures what you call LSR if it helps to distinguish between cells inside and outside LSR (lines 199-206).

This is a good idea and has been added onto the figures.

**References**

1.	Hagiwara M, Kawahara T, Arai F. Local streamline generation by mechanical oscillation in a microfluidic chip for noncontact cell manipulations Related Articles. Cit Appl Phys Lett. 2012;101: 74102. doi:10.1063/1.4746247

2.	Jeon S, Kim S, Ha S, Lee S, Kim E, Kim SY, et al. Magnetically actuated microrobots as a platform for stem cell transplantation. Sci Robot. 2019;4. doi:10.1126/scirobotics.aav4317

3.	Hu W, Fan Q, Ohta AT. An opto-thermocapillary cell micromanipulator. Lab Chip. 2013;13: 2285–2291. doi:10.1039/c3lc50389e

4.	Yeo LY, Friend JR. Ultrafast microfluidics using surface acoustic waves. Biomicrofluidics. American Institute of Physics Inc.; 2009. p. 012002. doi:10.1063/1.3056040

5.	Meng L, Cai F, Jiang P, Deng Z, Li F, Niu L, et al. On-chip targeted single cell sonoporation with microbubble destruction excited by surface acoustic waves. Appl Phys Lett. 2014;104: 073701. doi:10.1063/1.4865770

6.	Shi J, Ahmed D, Mao X, Lin SCS, Lawit A, Huang TJ. Acoustic tweezers: Patterning cells and microparticles using standing surface acoustic waves (SSAW). Lab Chip. 2009;9: 2890–2895. doi:10.1039/b910595f

7.	Bhagat AAS, Hou HW, Li LD, Lim CT, Han J. Pinched flow coupled shear-modulated inertial microfluidics for high-throughput rare blood cell separation. Lab Chip. 2011;11: 1870–1878. doi:10.1039/c0lc00633e

8.	Nieuwstadt HA, Seda R, Li DS, Fowlkes JB, Bull JL. Microfluidic particle sorting utilizing inertial lift force. Biomed Microdevices. 2011;13: 97–105. doi:10.1007/s10544-010-9474-6

9.	Grilli S, Ferraro P. Dielectrophoretic trapping of suspended particles by selective pyroelectric effect in lithium niobate crystals. Appl Phys Lett. 2008;92: 232902. doi:10.1063/1.2943319

10.	Srivastava SK, Gencoglu A, Minerick AR. DC insulator dielectrophoretic applications in microdevice technology: A review. Analytical and Bioanalytical Chemistry. 2011. pp. 301–321. doi:10.1007/s00216-010-4222-6

11.	Braff WA, Pignier A, Buie CR. High sensitivity three-dimensional insulator-based dielectrophoresis. Lab Chip. 2012;12: 1327–1331. doi:10.1039/c2lc21212a

12.	Hu W, Ishii KS, Ohta AT. Micro-assembly using optically controlled bubble microrobots. Appl Phys Lett. 2011;99: 094103. doi:10.1063/1.3631662

13.	Sun Y, Evans JS, Lee T, Senyuk B, Keller P, He S, et al. Optical manipulation of shape-morphing elastomeric liquid crystal microparticles doped with gold nanocrystals. Appl Phys Lett. 2012;100: 241901. doi:10.1063/1.4729143

14.	Onda K, Arai F. Multi-beam bilateral teleoperation of holographic optical tweezers. Opt Express. 2012;20: 3633. doi:10.1364/oe.20.003633

15.	Selman Sakar M, Steager EB, Hyung Kim D, Jun Kim M, Pappas GJ, Steager E, et al. Single Cell Manipulation using Ferromagnetic Composite Microtransporters. Appl Phys Lett. 2010;96: 43705. doi:10.1063/1.3293457

16.	Diller E, Floyd S, Pawashe C, Sitti M. Control of multiple heterogeneous magnetic microrobots in two dimensions on nonspecialized surfaces. IEEE Trans Robot. 2012;28: 172–182. doi:10.1109/TRO.2011.2170330

17.	Tottori S, Zhang L, Qiu F, Krawczyk KK, Franco-Obregõn A, Nelson BJ. Magnetic helical micromachines: Fabrication, controlled swimming, and cargo transport. Adv Mater. 2012;24: 811–816. doi:10.1002/adma.201103818

18.	Kim DH, Cheang UK, Khidai L, Byun D, Kim MJ. Artificial magnetotactic motion control of Tetrahymena pyriformis using ferromagnetic nanoparticles: A tool for fabrication of microbiorobots. Appl Phys Lett. 2010;97: 173702. doi:10.1063/1.3497275

19.	Akiyama Y, Iwabuchi K, Furukawa Y, Morishima K. Long-term and room temperature operable bioactuator powered by insect dorsal vessel tissue. Lab Chip. 2009;9: 140–144. doi:10.1039/b809299k

20.	Murayama Y, Constantinou CE, Omata S. Micro-mechanical sensing platform for the characterization of the elastic properties of the ovum via uniaxial measurement. J Biomech. 2004;37: 67–72. doi:10.1016/S0021-9290(03)00242-2

21.	Wakayama T, Perry ACF, Zuccotti M, Johnson KR, Yanagimachi R. Full-term development of mice from enucleated oocytes injected with cumulus cell nuclei. Nature. 1998;394: 369–374. doi:10.1038/28615

22.	Liu X, Fernandes R, Jurisicova A, Casper RF, Sun Y. In situ mechanical characterization of mouse oocytes using a cell holding device. Lab Chip. 2010;10: 2154. doi:10.1039/c004706f

23.	Bi C, Guix M, Johnson B, Jing W, Cappelleri D. Design of Microscale Magnetic Tumbling Robots for Locomotion in Multiple Environments and Complex Terrains. Micromachines. 2018;9: 68. doi:10.3390/mi9020068

24.	Huo B, Lu XL, Guo XE. Intercellular calcium wave propagation in linear and circuit-like bone cell networks. Philos Trans R Soc A Math Phys Eng Sci. 2010;368: 617–633. doi:10.1098/rsta.2009.0221

25.	Lu XL, Huo B, Chiang V, Guo XE. Osteocytic network is more responsive in calcium signaling than osteoblastic network under fluid flow. J Bone Miner Res. 2012;27: 563–574. doi:10.1002/jbmr.1474

26.	Middleton K, Al-Dujaili S, Mei X, Günther A, You L. Microfluidic co-culture platform for investigating osteocyte-osteoclast signalling during fluid shear stress mechanostimulation. J Biomech. 2017;59: 35–42. doi:10.1016/j.jbiomech.2017.05.012

27.	You L, Temiyasathit S, Lee P, Kim CH, Tummala P, Yao W, et al. Osteocytes as mechanosensors in the inhibition of bone resorption due to mechanical loading. Bone. 2008;42: 172–179. doi:10.1016/j.bone.2007.09.047

28.	Shah KM, Stern MM, Stern AR, Pathak JL, Bravenboer N, Bakker AD. Osteocyte isolation and culture methods. Bonekey Rep. 2016;5. doi:10.1038/bonekey.2016.65

29.	Middleton K, Kondiboyina A, Borrett M, Cui Y, Mei X, You L. Microfluidics approach to investigate the role of dynamic similitude in osteocyte mechanobiology. J Orthop Res. 2017;36: 663–671. doi:10.1002/jor.23773

30.	Weinbaum S, Cowin SC, Zeng Y. A model for the excitation of osteocytes by mechanical loading-induced bone fluid shear stresses. J Biomech. 1994;27: 339–360. doi:10.1016/0021-9290(94)90010-8

31.	Li J, Rose E, Frances D, Sun Y, You L. Effect of oscillating fluid flow stimulation on osteocyte mRNA expression. J Biomech. 2012;45: 247–251. doi:10.1016/j.jbiomech.2011.10.037

32.	Cresswell EN, Nguyen TM, Horsfield MW, Alepuz AJ, Metzger TA, Niebur GL, et al. Mechanically induced bone formation is not sensitive to local osteocyte density in rat vertebral cancellous bone. J Orthop Res. 2017;36: 672–681. doi:10.1002/jor.23606

33.	Tanaka-Kamioka K, Kamioka H, Ris H, Lim S-S. Osteocyte Shape Is Dependent on Actin Filaments and Osteocyte Processes Are Unique Actin-Rich Projections. J Bone Miner Res. 2009;13: 1555–1568. doi:10.1359/jbmr.1998.13.10.1555
